# Menopause-dependent correlations of blood mercury levels with bone mineral density and appendicular lean mass index in middle-aged women

**DOI:** 10.3389/fpubh.2024.1501162

**Published:** 2025-02-19

**Authors:** Feng Xu, Yanfei Wang, Jinqiong Fang, Zhongxin Zhu

**Affiliations:** ^1^Department of Clinical Laboratory, The First People’s Hospital of Xiaoshan District, Xiaoshan Affiliated Hospital of Wenzhou Medical University, Hangzhou, Zhejiang, China; ^2^Department of Hospital Management, The First People’s Hospital of Xiaoshan District, Xiaoshan Affiliated Hospital of Wenzhou Medical University, Hangzhou, Zhejiang, China; ^3^Department of Osteoporosis Care and Control, The First People’s Hospital of Xiaoshan District, Xiaoshan Affiliated Hospital of Wenzhou Medical University, Hangzhou, Zhejiang, China

**Keywords:** blood mercury levels, bone mineral density, appendicular lean mass index, menopausal status, environmental exposure

## Abstract

**Background:**

Mercury exposure poses significant health risks, yet its effects on bone mineral density (BMD) and appendicular lean mass index (ALMI) in middle-aged women remain poorly understood. This study aimed to investigate the associations between blood mercury levels (BML) and these key indicators of skeletal health and body composition, with special attention to the potential modifying effect of menopausal status.

**Methods:**

We analyzed data from 1,648 women aged 40–59 years (782 premenopausal, 866 postmenopausal) using the National Health and Nutrition Examination Survey (NHANES) 2011–2018. Multiple linear regression models were employed to assess the relationships between LnBML and both lumbar BMD and ALMI, adjusting for relevant covariates.

**Results:**

There was complex, non-linear associations between LnBML and skeletal health parameters. Notably, the relationship between LnBML and lumbar BMD differed significantly based on menopausal status (*P* for interaction <0.001). In premenopausal women, LnBML was negatively associated with BMD (*β* = −0.018, 95% CI: −0.029, −0.007), while in postmenopausal women, a positive association was observed (*β* = 0.025, 95% CI: 0.014, 0.036). Conversely, LnBML demonstrated a significant positive association with ALMI (*β* = 0.054, 95% CI: 0.025, 0.083, *p* < 0.001) in the fully adjusted model.

**Conclusion:**

Our findings revealed intricate, menopause-dependent relationships between BML and skeletal health parameters in middle-aged women. These results underscore the complex interplay between environmental exposures and women’s health across the menopausal transition, highlighting the need for further research to elucidate underlying mechanisms and inform targeted interventions.

## Introduction

Mercury, a pervasive and highly toxic heavy metal, ranks among the top three priority substances of global public health concern due to its profound impacts on human health and ecosystems (1). In the general population, exposure to mercury predominantly transpires via three principal avenues: ingestion of dietary sources, notably the consumption of fish and seafood contaminated with methylmercury; occupational hazards, encompassing dental amalgams and industrial activities; and environmental contamination, such as polluted air and tainted water (2, 3). Moreover, the speciation of mercury—elemental, inorganic, or organic—profoundly affects its toxicokinetic properties and the health outcomes associated with exposure (4). These findings carry particularly alarming implications for vulnerable populations, underscoring the urgent need for stringent global policies to mitigate mercury pollution (5).

Concurrently, bone mineral density (BMD) serves as a critical indicator of skeletal integrity and a primary predictor of osteoporosis risk (6). The perimenopausal transition in women is characterized by accelerated bone loss, significantly increasing their susceptibility to fractures and osteoporosis (7). This life stage is further marked by notable changes in body composition, particularly in appendicular lean mass (8). The appendicular lean mass index (ALMI), a valuable measure of skeletal muscle mass, has been closely associated with sarcopenia and functional capacity (9, 10).

Emerging evidence suggests that environmental factors, including exposure to heavy metals, may exert substantial influence on bone metabolism and body composition (11–13). However, the specific relationship between blood mercury levels (BML) and skeletal health parameters remains understudied, representing a critical gap in our understanding of environmental toxicology and women’s health. Therefore, this study aimed to elucidate the association between BML, BMD and ALMI in middle-aged women using large-scale population data, with particular focus on examining whether these relationships differ by menopausal status. Our findings may have important implications for public health policies, environmental regulations, and clinical practices related to women’s health and aging.

## Methods

### Study design and population

This study analyzed data from the National Health and Nutrition Examination Survey (NHANES), a comprehensive, ongoing cross-sectional survey designed to assess the health and nutritional status of adults and children in the United States. The NHANES protocol was approved by the ethics review board of the National Center for Health Statistics, with all participants providing written informed consent.

We extracted data from the NHANES database spanning 2011 to 2018, focusing on women aged 40–59 years (*n* = 3,908). After excluding participants with missing data for BML (*n* = 1,240), lumbar BMD (*n* = 645), ALMI (*n* = 243), and those with indeterminate menstrual status (*n* = 132), the final cohort comprised 1,648 participants (782 premenopausal and 866 postmenopausal women).

### Study variables

The primary exposure variable was BML, measured using inductively coupled plasma mass spectrometry (ICP-MS) with quadrupole technology. BML data underwent natural log-transformation (Ln) to approximate a normal distribution. Outcome variables included lumbar BMD and ALMI, both measured via dual-energy X-ray absorptiometry. ALMI was calculated as appendicular lean mass [g] divided by height squared [m^2^]. Covariates encompassed both categorical variables (race, educational level, menstrual status, history of hypertension, diabetes, cancer or malignancy, and moderate recreational activities) and continuous variables (age, body mass index [BMI], total protein, blood urea nitrogen, serum uric acid, and serum calcium). Detailed methodologies for BML, BMD, and ALMI measurements are available at wwwn.cdc.gov/nchs/nhanes/.

### Statistical analyses

All analyses utilized weighted NHANES samples to ensure national representativeness. To examine the independent associations of BML with BMD and ALMI, we employed weighted multiple linear regression models. Following the statement of Strengthening the Reporting of Observational Studies in Epidemiology (STROBE) guidelines (14), we constructed four models: Model 1 (unadjusted), Model 2 (adjusted for age and race), Model 3 (adjusted for age, race, and BMI), and Model 4 (fully adjusted model including all screened covariates). Smooth curve fittings and generalized additive models were applied to explore potential non-linear relationships.

Continuous variables were expressed as mean ± standard deviation, while categorical variables were presented as weighted percentages. Statistical significance was set at *p* < 0.05. All analyses were performed using Empowerstats^1^ and R software (version 3.4.3). Statistical significance was set at *p* < 0.05.

## Results

### Participant characteristics

Table 1 presents participant characteristics stratified by menopausal status. Postmenopausal women exhibited higher prevalence of hypertension, diabetes, and cancer, alongside elevated levels of blood urea nitrogen, serum uric acid, and serum calcium. Notably, postmenopausal women demonstrated significantly lower lumbar BMD and ALMI compared to their premenopausal counterparts.

**Table 1:** The characteristics of participants according to menstrual status.

	Premenopausal women (*n* = 782)	Postmenopausal women (*n* = 866)	*p*-value
Age (years)	46.1 ± 4.1	52.8 ± 5.0	<0.001
Race/Ethnicity (%)			0.408
Non-Hispanic White	63.0	66.1	
Non-Hispanic Black	11.5	11.7	
Mexican American	9.2	7.4	
Other race/ethnicity	16.3	14.8	
Education level (%)			<0.001
Less than high school	12.2	13.0	
High school	15.3	22.4	
More than high school	72.4	64.5	
Moderate recreational activities (%)			0.047
Yes	50.9	46.0	
No	49.1	54.0	
Hypertension (%)			<0.001
Yes	23.4	35.2	
No	76.6	64.8	
Diabetes (%)			0.004
Yes	6.1	11.4	
No	93.9	88.6	
Cancer or malignancy (%)			<0.001
Yes	6.6	11.4	
No	93.4	88.6	
Body mass index (kg/m^2^)	29.2 ± 7.3	29.3 ± 6.8	0.857
Total protein (g/L)	70.3 ± 4.3	70.7 ± 4.5	0.061
Blood urea nitrogen (mmol/L)	4.1 ± 1.3	4.8 ± 1.7	<0.001
Serum uric acid (μmol/L)	268.1 ± 61.6	286.6 ± 71.1	<0.001
Serum calcium (mmol/L)	2.3 ± 0.1	2.4 ± 0.1	<0.001
Blood mercury (nmol/L)	7.2 ± 9.7	7.6 ± 11.7	<0.001
Lumbar bone mineral density (g/cm^2^)	1.1 ± 0.1	1.0 ± 0.2	<0.001
Appendicular lean mass index (g/m^2^)	7042.8 ± 1401.1	6847.1 ± 1300.7	<0.001

Mean ± SD for continuous variables: *P*-value was calculated by weighted linear regression model.

% for categorical variables: *P*-value was calculated by weighted chi-square test.

### Associations between BML, BMD, and ALMI

**Figure 1:**
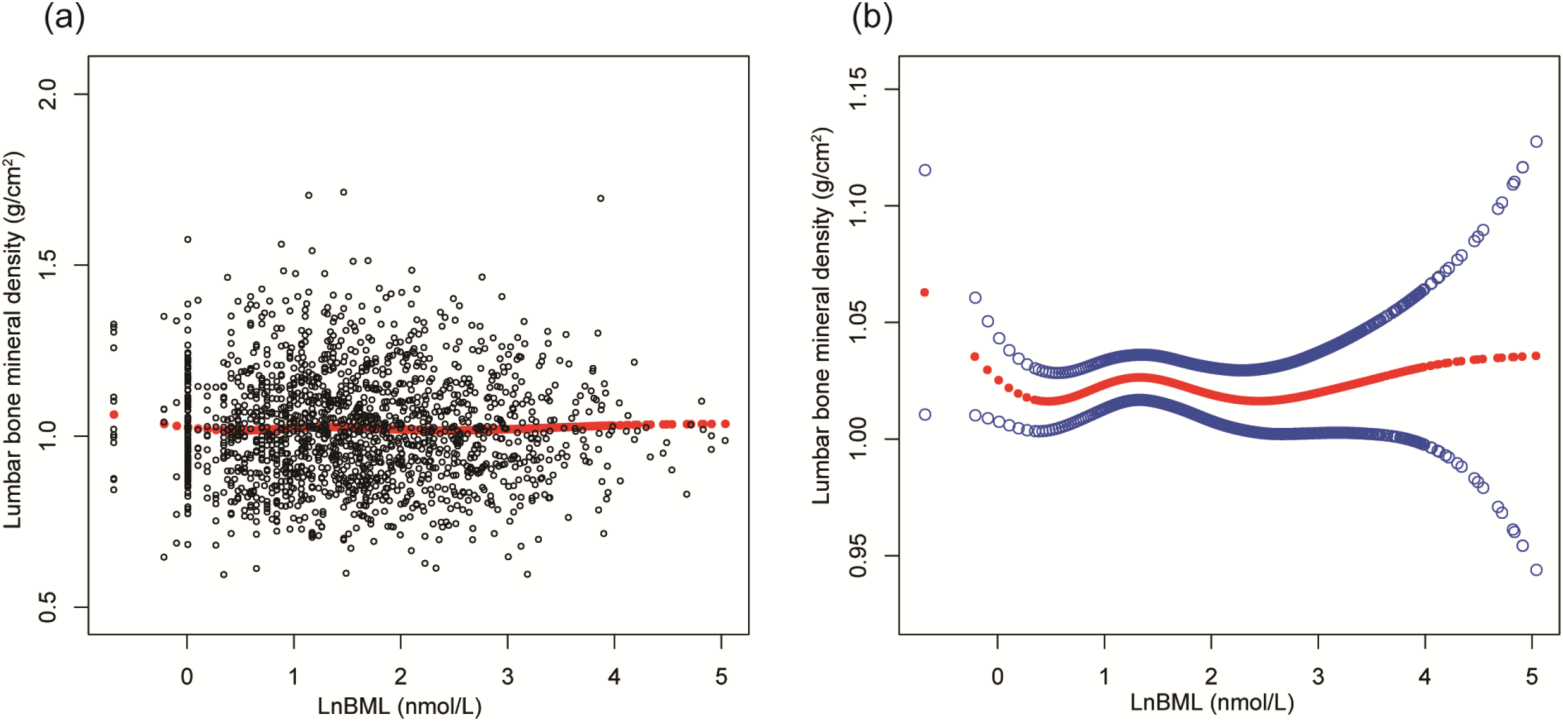
The association between LnBML and lumbar BMD (g/cm^2^). **(A)** Each black point represents a sample. **(B)** Solid red line represents the smooth curve fit between variables. Blue bands represent the 95% of confidence interval from the fit. Age, race, educational level, menstrual status, history of hypertension, history of diabetes, history of cancer or malignancy, body mass index, moderate recreational activities, total protein, blood urea nitrogen, serum uric acid, and serum calcium were adjusted. BML, blood mercury levels; BMD, bone mineral density.

**Figure 2:**
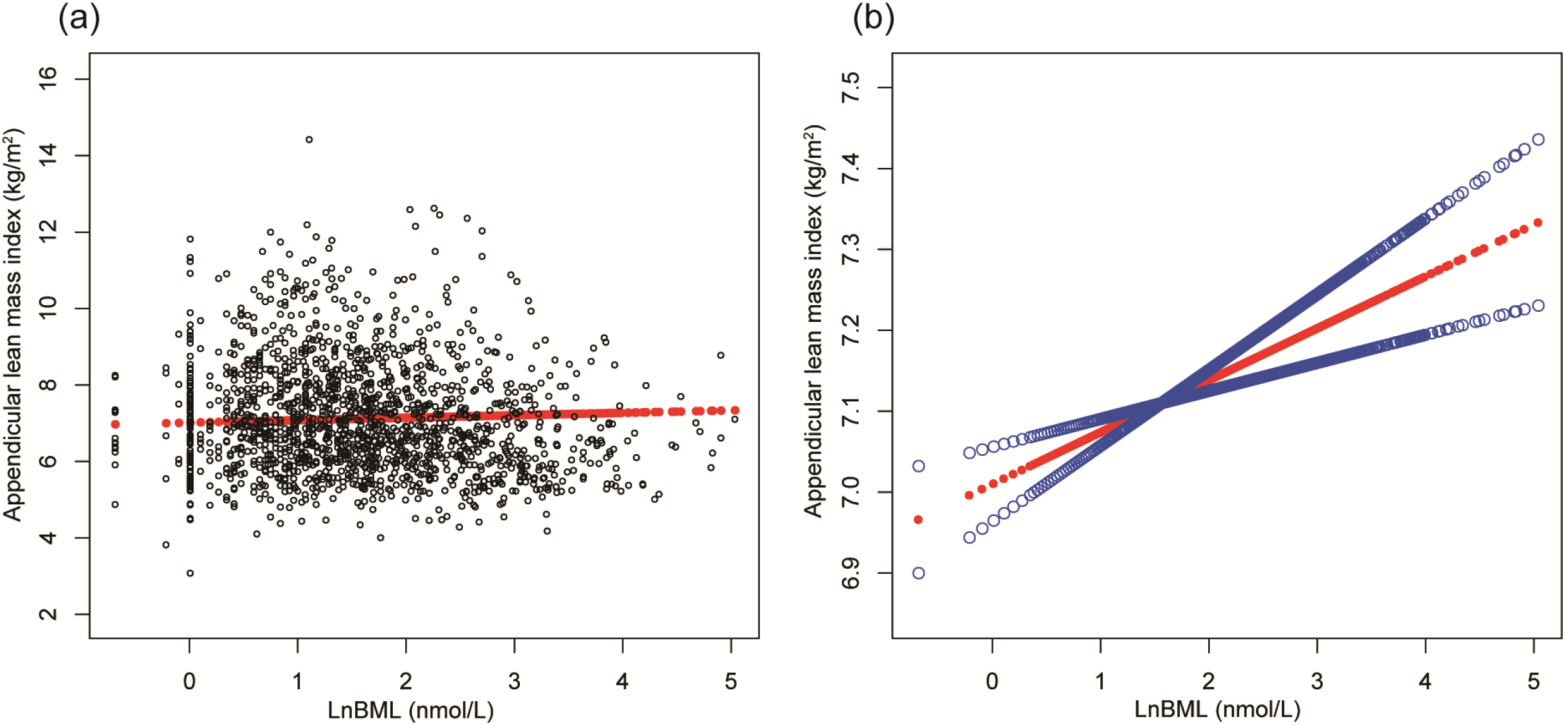
The association between LnBML and ALMI (g/m^2^). **(A)** Each black point represents a sample. **(B)** Solid red line represents the smooth curve fit between variables. Blue bands represent the 95% of confidence interval from the fit. Age, race, educational level, menstrual status, history of hypertension, history of diabetes, history of cancer or malignancy, body mass index, moderate recreational activities, total protein, blood urea nitrogen, serum uric acid, and serum calcium were adjusted. BML, blood mercury levels; ALMI, appendicular lean mass index.

LnBML exhibited complex associations with BMD and ALMI (Table 2). In fully adjusted models (Model 4), LnBML showed no significant association with BMD (*β* = 0.006, 95% CI: −0.002, 0.014). However, LnBML quartiles revealed a non-linear relationship with BMD, albeit without statistical significance (*p* for trend = 0.516). Conversely, LnBML demonstrated a significant positive association with ALMI (β = 0.054, 95% CI: 0.025, 0.083, *p* < 0.001). This relationship was corroborated by a significant trend across LnBML quartiles (*p* for trend = 0.038), with the highest quartile showing the strongest association. These non-linear and positive relationships were further substantiated by smooth curve fittings (Figures 1, 2).

**Table 2:** Association of LnBML with BMD and ALMI.

	Model 1 β (95% CI)	Model 2 β (95% CI)	Model 3 β (95% CI)	Model 4 β (95% CI)
BMD (g/cm^2^)	0.002 (−0.006, 0.010)	0.010 (0.002, 0.017)*	0.011 (0.003, 0.019)**	0.006 (−0.002, 0.014)
LnBML Q4				
Q1	Reference	Reference	Reference	Reference
Q2	0.017 (−0.004, 0.038)	0.016 (−0.004, 0.036)	0.015 (−0.005, 0.036)	0.011 (−0.009, 0.031)
Q3	−0.011 (−0.032, 0.010)	−0.002 (−0.022, 0.018)	−0.001 (−0.021, 0.019)	−0.014 (−0.034, 0.006)
Q4	0.008 (−0.014, 0.030)	0.028 (0.007, 0.049)	0.031 (0.010, 0.052)	0.017 (−0.004, 0.039)
*P* for trend	0.896	0.072	0.035	0.516
ALMI (kg/m^2^)	−0.123 (−0.189, −0.057)***	−0.102 (−0.166, −0.038)**	0.069 (0.040, 0.097)***	0.054 (0.025, 0.083)***
LnBML Q4				
Q1	Reference	Reference	Reference	Reference
Q2	0.312 (0.132, 0.492)	0.243 (0.074, 0.413)	0.121 (0.046, 0.196)	0.104 (0.029, 0.178)
Q3	−0.074 (−0.250, 0.103)	−0.094 (−0.261, 0.074)	0.076 (0.002, 0.150)	0.047 (−0.028, 0.121)
Q4	−0.305 (−0.489, −0.120)	−0.258 (−0.435, −0.080)	0.148 (0.069, 0.227)	0.107 (0.026, 0.189)
*P* for trend	<0.001	<0.001	0.001	0.038

Model 1: no covariates were adjusted.

Model 2: age and race were adjusted.

Model 3: age, race and body mass index were adjusted.

Model 4: age, race, educational level, menstrual status, history of hypertension, history of diabetes, history of cancer or malignancy, body mass index, moderate recreational activities, total protein, blood urea nitrogen, serum uric acid, and serum calcium were adjusted.

^*^p < 0.05, ^**^p < 0.01, ^***^p < 0.001.

BML, blood mercury levels; BMD, bone mineral density; ALMI, appendicular lean mass index.

### Subgroup analyses

**Figure 3:**
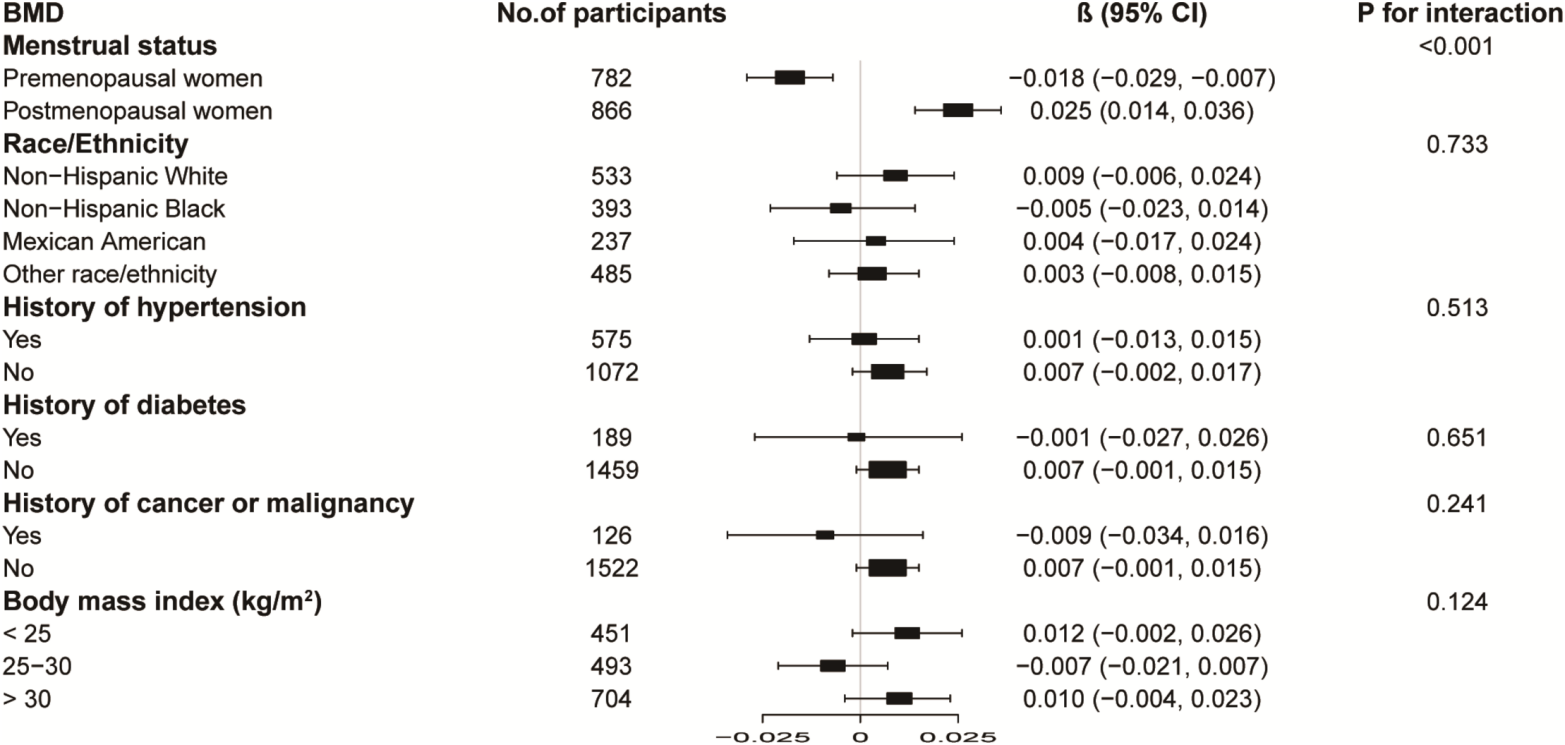
Subgroup analysis of the association between LnBML and lumbar BMD (g/cm^2^). Age, race, educational level, menstrual status, history of hypertension, history of diabetes, history of cancer or malignancy, body mass index, moderate recreational activities, total protein, blood urea nitrogen, serum uric acid, and serum calcium were adjusted. In the subgroup analysis, the model is not adjusted for the stratification variable itself. BML, blood mercury levels; BMD, bone mineral density.

**Figure 4:**
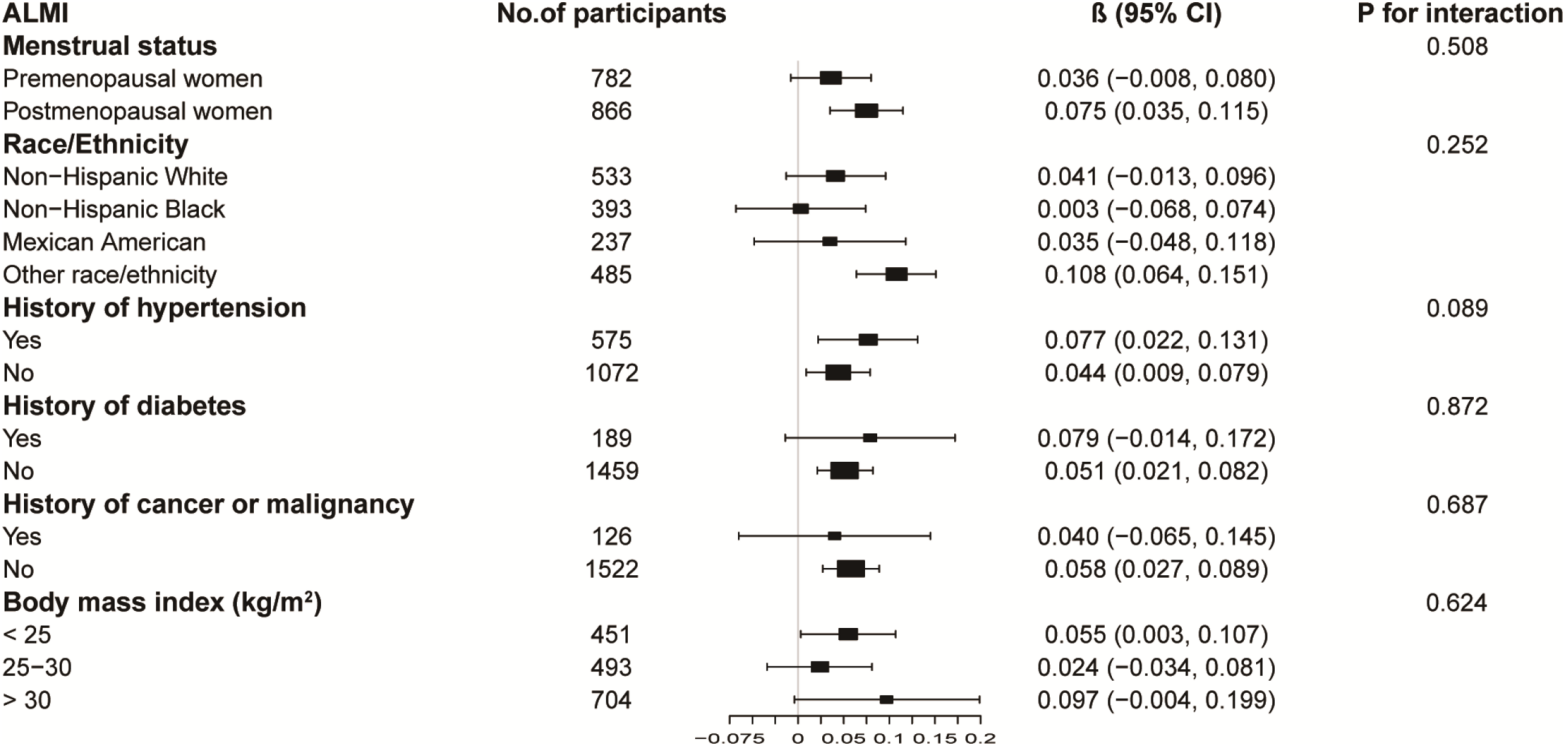
Subgroup analysis of the association between LnBML and ALMI (g/m^2^). Age, race, educational level, menstrual status, history of hypertension, history of diabetes, history of cancer or malignancy, body mass index, moderate recreational activities, total protein, blood urea nitrogen, serum uric acid, and serum calcium were adjusted. In the subgroup analysis, the model is not adjusted for the stratification variable itself. BML, blood mercury levels; ALMI, appendicular lean mass index.

Figures 3, 4 illustrate subgroup analyses of LnBML correlations with lumbar BMD and ALMI across various demographic and health-related strata. Notably, the relationship between LnBML and BMD exhibited a significant dichotomy based on menopausal status (*p* for interaction <0.001). A negative correlation was observed in premenopausal women (*β* = −0.018, 95% CI: −0.029, −0.007), contrasting with a positive correlation in postmenopausal women (*β* = 0.025, 95% CI: 0.014, 0.036). The association between LnBML and ALMI demonstrated a positive trend, reaching statistical significance in postmenopausal women (*β* = 0.075, 95% CI: 0.035, 0.115).

**Figure 5:**
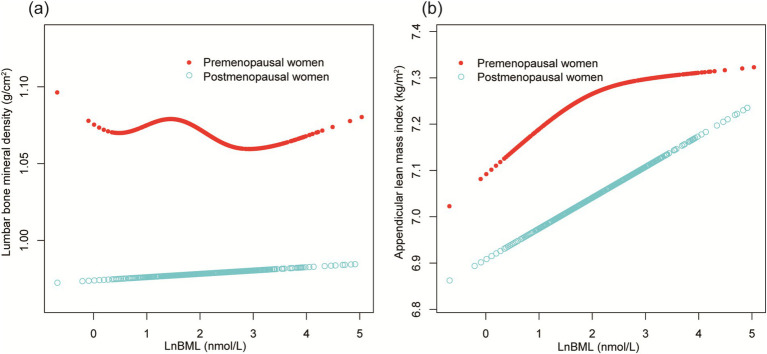
The association of LnBML with lumbar BMD (g/cm^2^) and ALMI (g/m^2^), stratified by menstrual status. **(A)** The association between LnBML and lumbar BMD. **(B)** The association between LnBML and ALMI. Age, race, educational level, history of hypertension, history of diabetes, history of cancer or malignancy, body mass index, moderate recreational activities, total protein, blood urea nitrogen, serum uric acid, and serum calcium were adjusted. BML, blood mercury levels; BMD, bone mineral density; ALMI, appendicular lean mass index.

Figure 5 further confirms these menstrual status-stratified associations between LnBML and both lumbar BMD and ALMI through smooth curve fittings and generalized additive models.

## Discussion

Our findings revealed complex, non-linear relationships between BML, lumbar BMD, and ALMI in middle-aged women, with notable differences based on menopausal status. The significant interaction between BML and menopausal status for BMD emerged as a key finding, demonstrating that the impact of environmental mercury exposure on musculoskeletal health fundamentally differs across the menopausal transition.

Evidence suggests that there is a complex relationship between mercury exposure and osteoporosis. A groundbreaking study in Korea (15) revealed a counterintuitive protective effect of elevated BML against osteoporosis in postmenopausal women, suggesting a nuanced interplay between mercury exposure and bone health. Contrastingly, research in Spain (16) found no significant correlations between dietary mercury intake and bone health parameters in premenopausal women, highlighting the potential importance of exposure route and physiological state. Our findings align with and extend these observations, revealing a striking interaction between BML and menopausal status that suggests a fundamental shift in mercury-bone interactions across the menopausal transition.

Further complicating this picture, a recent analysis of NHANES data (17) demonstrated a negative association between BML and total BMD in female adolescents, underscoring the age-dependent nature of mercury’s impact on bone metabolism. Our findings align with and extend these observations, revealing a striking interaction between BML and menopausal status. We observed diametrically opposed correlations in premenopausal and postmenopausal women, a discovery that not only corroborates previous studies but also suggests a fundamental shift in mercury-bone interactions across the menopausal transition.

The mechanistic underpinnings of mercury’s effects on bone metabolism remain elusive and controversial. Short-term mercury exposure studies have yielded conflicting results: one investigation (18) reported enhanced osteoclast activity and increased blood calcium levels in fish scales, while a subsequent experiment (19) observed reduced osteoclast activity and potential osteoblast protection. These contradictory findings underscore the complexity of mercury’s biological effects and the need for further research to elucidate the precise molecular pathways involved.

The well-established link between estrogen deficiency and increased osteoporosis risk (20, 21) provides a potential framework for understanding our observations. Intriguingly, previous research has demonstrated that mercuric chloride can exert estrogen-like effects on osteoblast activity (22). This phenomenon may offer a plausible explanation for the differential effects of BML on BMD observed in our menstrual status-stratified analysis.

The relationship between mercury exposure and its effects on lean mass and sarcopenia remains largely unexplored, representing a significant gap in our understanding of environmental toxicants and musculoskeletal health. While direct evidence is limited, the established similarities in pathophysiology and risk factors between osteoporosis and sarcopenia (23–25) provide a compelling rationale for investigating potential associations between chronic mercury exposure and muscle-related outcomes.

Our study presents novel findings that contribute to this emerging field of inquiry. Initially, our unadjusted models (Models 1 and 2) aligned with previous research from the Korea National Health and Nutritional Examination Surveys, which reported an increased prevalence of sarcopenia in older adult populations with elevated BML (26). However, upon adjusting for BMI in Models 3 and 4, we observed a striking reversal in this relationship. This unexpected shift underscores the complex interplay between mercury exposure and muscle mass, highlighting the critical importance of considering confounding factors in environmental health research. Future studies should aim to elucidate the underlying biological mechanisms, explore dose–response relationships, and investigate potential interactions with other environmental toxicants and lifestyle factors.

Our study, while illuminating the intricate relationships between BML, BMD, and ALMI in middle-aged women, is constrained by several methodological limitations. First, the cross-sectional nature of our NHANES-based analysis precludes causal inferences, necessitating longitudinal investigations to establish temporal relationships. Second, we relied solely on total blood mercury measurements without detailed information about exposure sources or mercury species. Future studies would benefit from collecting comprehensive exposure data, including dietary patterns, occupational history, and environmental exposure assessments, to better understand the relationship between specific mercury exposure pathways and skeletal health outcomes. Third, despite rigorous covariate adjustment, residual confounding from unmeasured variables such as occupational exposures, genetic polymorphisms, and hormonal factors cannot be ruled out. Fourth, the research was confined to middle-aged women aged 40–59 years, which may limit the generalizability of our findings. Future studies should explore mercury’s effects across broader age ranges and include male populations to provide a more comprehensive understanding of environmental mercury exposure’s impact on skeletal health. Lastly, while we identify significant associations between BML and musculoskeletal parameters, the underlying biological mechanisms remain elusive. The observed differential effects based on menopausal status underscore the necessity for mechanistic studies to elucidate the molecular pathways mediating mercury’s impact on skeletal health.

## Conclusion

Our study unveils complex, menopause-dependent relationships between BML and skeletal health parameters in middle-aged women. The striking dichotomy in BML-BMD correlations between pre- and post-menopausal women highlights the critical role of menopausal status in modulating environmental mercury’s effects on bone health. These findings emphasize the need for targeted interventions and further research to understand the underlying mechanisms of these relationships across the menopausal transition.

## Data Availability

The raw data supporting the conclusions of this article will be made available by the authors, without undue reservation.

## Footnotes

^1^ www.empowerstats.com
